# Tactile Stimulation of the Face and the Production of Facial Expressions Activate Neurons in the Primate Amygdala

**DOI:** 10.1523/ENEURO.0182-16.2016

**Published:** 2016-10-07

**Authors:** Clayton P. Mosher, Prisca E. Zimmerman, Andrew J. Fuglevand, Katalin M. Gothard

**Affiliations:** Department of Physiology, College of Medicine, The University of Arizona, Tucson, AZ, USA

**Keywords:** emotion, face, monkey, social communication, somatosensory, touch

## Abstract

The majority of neurophysiological studies that have explored the role of the primate amygdala in the evaluation of social signals have relied on visual stimuli such as images of facial expressions. Vision, however, is not the only sensory modality that carries social signals. Both humans and nonhuman primates exchange emotionally meaningful social signals through touch. Indeed, social grooming in nonhuman primates and caressing touch in humans is critical for building lasting and reassuring social bonds. To determine the role of the amygdala in processing touch, we recorded the responses of single neurons in the macaque amygdala while we applied tactile stimuli to the face. We found that one-third of the recorded neurons responded to tactile stimulation. Although we recorded exclusively from the right amygdala, the receptive fields of 98% of the neurons were bilateral. A fraction of these tactile neurons were monitored during the production of facial expressions and during facial movements elicited occasionally by touch stimuli. Firing rates arising during the production of facial expressions were similar to those elicited by tactile stimulation. In a subset of cells, combining tactile stimulation with facial movement further augmented the firing rates. This suggests that tactile neurons in the amygdala receive input from skin mechanoceptors that are activated by touch and by compressions and stretches of the facial skin during the contraction of the underlying muscles. Tactile neurons in the amygdala may play a role in extracting the valence of touch stimuli and/or monitoring the facial expressions of self during social interactions.

## Significance Statement

The primate amygdala receives sensory inputs of all modalities, yet remarkably little is known about how the amygdala may process touch. Here we report for the first time that neurons in the monkey amygdala respond to touching of the face. Similar to visually responsive neurons, tactile neurons in the amygdala may be involved in extracting the positive or negative valence of touch stimuli. The activity of these neurons was modulated during the production of facial expressions and the contraction of facial muscles, suggesting that these neurons can also signal the expressive status of the face. The presence of touch-sensitive neurons in the primate amygdala expands the known functions of this important structure for emotion and for social communication with facial expressions.

## Introduction

Among the canonical senses, touch provides a basic means of social contact and can convey both positive and negative emotions. Indeed, a primate’s earliest socioemotional experience may be the protective embrace of a parent. Touch is used by both humans and nonhuman primates to build and maintain social bonds throughout life, yet little is known about how brain structures involved in evaluating socioemotional stimuli, such as the amygdala, process tactile signals. Only a few rodent studies showed a convergence of tactile and auditory inputs on single neurons in the amygdala (Romanski et al., 1993; Windels et al., 2016). A small number of recent imaging studies suggest that the human amygdala processes social and affective touch (Gordon et al., 2013; McGlone et al., 2014; Morrison, 2016), but the neural activity associated with these hemodynamic changes remains unknown.

The responses of neurons in the primate brain to socioemotional stimuli have been explored almost exclusively in the visual domain (Plakke and Romanski, 2016), as vision is the dominant sensory modality for social communication among primates (Adolphs, 2010). Indeed, neurons in the human and nonhuman primate amygdala respond selectively to visual stimuli of social significance, including identity (Gothard et al., 2007), facial expressions (Gothard et al., 2007; Rutishauser et al., 2011), and eye contact (Mosher et al., 2014). Considerably less emphasis has been placed on auditory responses in the primate amygdala (Kuraoka and Nakamura, 2007; Resnik and Paz, 2015), and no reports are available on single-unit activity elicited by tactile stimuli. The scarcity of neurophysiological studies in the somatosensory domain is surprising given the rich array of highly processed sensory information that multiple cortical areas send to the amygdala (Amaral et al., 1992). Indeed, the amygdala is one of the main hubs of the primate brain, where multiple processing pathways converge (Tomasi and Volkow, 2011). In the amygdala, the emotional and social significance of sensory inputs are evaluated through a sequence of processing stages localized to distinct nuclei. The outcome of this evaluation is forwarded to cortical (Amaral and Price, 1984; Cho et al., 2013) and subcortical (Price and Amaral, 1981; Gordon et al., 2013) targets.

Given the richness of emotional and social signals carried by touch, it seems likely that neurons in the amygdala respond to tactile stimulation. Here we present evidence that the activation of mechanoceptors in the facial skin, either by touch or by facial movements, increases activity in a subset of amygdala neurons. In this study, we focused on responses to facial stimulation because (1) the face has the largest representation in the cortical somatosensory maps (Kaas, 1993), (2) the facial skin is a target of social grooming, and (3) the face is a key somatic substrate through which social information is broadcast to others via facial expressions.

## Materials and Methods

### Surgical procedures

All procedures on the animals were performed in accordance with The University of Arizona Animal Care Committee regulations. This study included three adult male macaques, Q, M, and Z (weight 7–12 kg; age 5–13 years). The stereotaxic coordinates of the right amygdala in each subject were calculated based on high-resolution (isotropic voxel size = 0.5 mm) structural magnetic resonance imaging (MRI) scans. Each monkey was implanted with a chamber mounted on the skull above the right amygdala. The implant consisted of a recording chamber and three titanium posts (Thomas Recording, Giessen, Germany) that could be used to immobilize the head. A craniotomy (∼13-mm diameter) was opened in the center of each chamber. Between recording sessions, the craniotomy was sealed with a silicone elastomer to maintain sterility and prevent scarring of the dura (Spitler et al., 2008).

### Electrophysiological equipment

Single-unit activity from the amygdala of each monkey was recorded acutely with a seven-channel Eckhorn drive (Thomas Recording) that advanced seven microelectrodes (quartz-glass insulated tungsten electrodes, 80- to 100-μm diameter, 1- to 2-MΩ impedance) into the right amygdala. The anatomical location of each electrode tip in the amygdala was calculated based on a postsurgical MRI scan with a cylinder of Vitamin E fish oil aligned in the chamber and used as contrast to visualize the trajectory of the electrodes. Single-unit activity was preamplified 20× (Thomas Recording), filtered (600–6000 Hz), amplified (1000×, Lynx-8; Neuralynx, Bozeman, MT), sampled continuously, and digitized at 40 kHz (Power 1401, Cambridge Electronic Design, Cambridge, UK). Single units were sorted offline using the Spike 2 template-matching algorithm and principal component analysis (Cambridge Electronic Design).

### Stimuli and recording procedure

Microelectrodes were independently advanced along the dorsoventral axis of the amygdala. When a well-isolated single unit was identified, the monkey was blindfolded to eliminate the possibility that neurons recorded during tactile stimulation were visually responsive. Next, the cell was tested with an array of tactile stimuli applied to the face including brushing, touching with a rigid blunt probe, and touching or stretching the skin with the fingertips. If the stability of the recording was maintained during this cursory tactile stimulation, the receptive field of the neuron was mapped by touching different facial regions multiple times. Tactile stimulation was applied to seven nonoverlapping facial regions: (1) nose, (2) area around the eyes, (3) muzzle, (4) mouth (including lips, tongue, teeth), (5) chin, (6) ears, and (7) cheeks. In addition, if a neuron had a receptive field in the perioral area, several objects, such as juice-filled syringes, were placed between the lips to evoke puckering and sucking movements. The monkeys were highly adapted to having their faces touched and typically did not make facial expressions or other facial movements during the application of touch stimuli. On occasion, when objects such as syringes were placed between their lips, monkeys puckered their lips in an attempt to hold and orally explore the object. These trials were analyzed separately.

Once neural responses to tactile stimuli were recorded, the blindfold was removed and the monkeys were provoked to make facial expressions. This was possible only when recording stability was maintained throughout the tactile stimulation and during the production of facial expressions. The stimuli used to elicit facial expressions included videos of conspecifics displayed on a computer monitor or mobile device, looming objects, eye contact with a human intruder, and enticement with fruits and nuts. The most commonly evoked expressions were lip smacking and bared-teeth displays (fear grimace), although on occasion, the monkeys also produced open-mouth threats and yawns. A video camera with a temporal resolution of 30 frames/s recorded the tactile stimulation and facial expressions of the monkeys. Given this frame rate, the temporal resolution for the onset of facial expressions was 33 ms. Video data were recorded simultaneously with neural data using the data acquisition system (Cambridge Electronic Design). The onset of facial expressions was marked as the earliest frame in which the monkey visibly moved his face.

### Histology

Recording sites were calculated based on MRI scans and confirmed, postmortem, by histological analysis of the brain of one monkey (Q). This brain was sectioned in the coronal plane in 40-μm slices, mounted on microscopic slides, and stained with Cresyl Violet. An electrolytic lesion was made during the final experiment (100-μA direct current pulse, 20-s duration), which was used to localize the electrode tips within the amygdala.

### Data analysis

All analyses were carried out using custom-designed programs in Matlab R2016 (MathWorks, Natick, MA). For statistical comparisons, Wilcoxon rank-sum or Kruskal–Wallis tests were used (nonparametric versions of the classic *t* test and ANOVA, respectively). Nonparametric tests were used because firing rates in the amygdala are typically low and not normally distributed. Statistical definition of tactile cells involved the following procedures. Single-unit firing was binned at 100-ms resolution and calculated in each bin with a 20-ms sliding window. This method has been successfully used for the analysis of neurons with low firing rates (Kennerley et al., 2009; Rudebeck et al., 2013). A Wilcoxon rank-sum test compared the spike rates during tactile stimulation to the spike rate during a pretouch baseline. If the activity in any bin during the tactile response window (0–500 ms after stimulus onset) was significantly different from baseline (500- to 1000-ms window preceding the stimulus), the cell was classified as tactile responsive (α = 0.05, Bonferroni corrected for number of bins: 0.05/20 bins = 0.0025). Once a cell was identified as being tactile responsive, its receptive field was identified using a Kruskal–Wallis test that compared the mean firing rate during stimulation in seven different facial areas. If the Kruskal–Wallis test was significant (α = 0.05), the cell was classified as having a receptive field. Post hoc Tukey–Kramer tests identified which areas of the face were within the neuron’s receptive field. On occasion, recording stability was lost before all the areas of the face were touched. Receptive field size was defined as the number of responsive areas divided by the number of tested areas. To determine whether the receptive fields were bilateral, we performed analyses at both single-cell and population levels. At the single-cell level, we compared the mean firing rate during stimulation of the left and right side of the face using a paired rank-sum test (α = 0.05). At the population level, we normalized the firing rate of all tactile cells by calculating the mean firing rate during stimulation on the left side and the right side of the face independently and then dividing by the maximum of the two. We then tested the population response to left- and right-side stimulation using a paired rank-sum test (α = 0.05). We also computed an average population response from the normalized firing rates of the 45 tactile neurons. For each cell, the peristimulus time histogram (PSTH) was scaled from 0 to 1, the minimum and maximum firing rate achieved in the binned PSTH.

## Results

Over the course of 34 sessions, we recorded 133 neurons from the right amygdalae of three adult male Rhesus monkeys: M, 22 neurons from 10 sessions; Q, 71 neurons from 15 sessions; and Z, 40 neurons from 9 sessions. On average, four neurons were recorded during each session. From our sample of 133 neurons, 45 (34%) met our criteria for tactile responsiveness.

### Characteristics of receptive fields of tactile-responsive neurons in the amygdala

**Figure 1.**
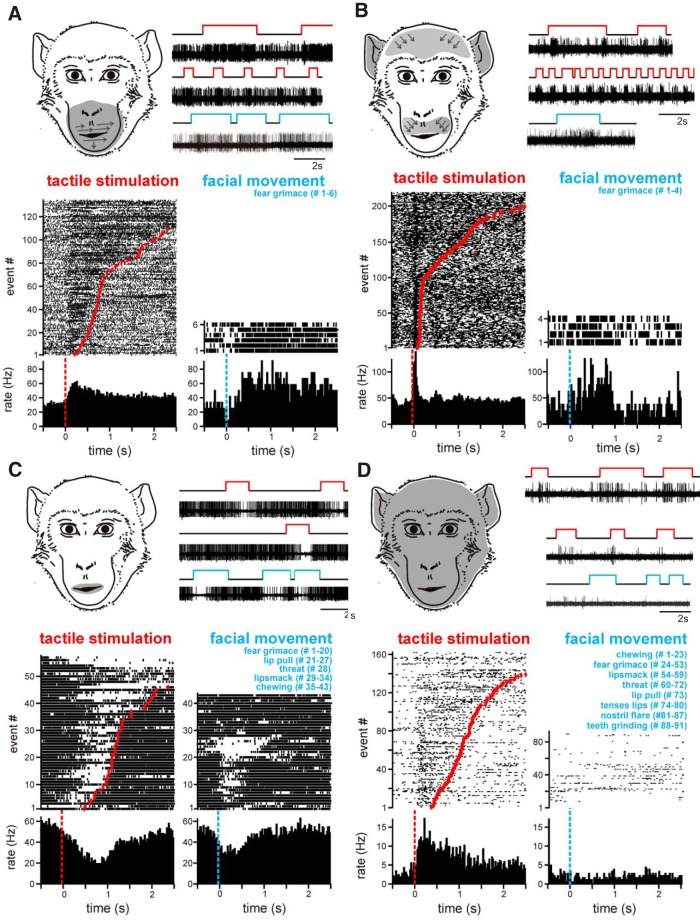
Example tactile neurons. Each panel shows the firing rate of a neuron during somatosensory stimulation of the face. The receptive fields are shaded in gray on the schematic drawing of the monkey face. The small arrows inside the receptive fields indicate the preferred direction of stimulation, if any. Raw traces of single-unit activity are shown to the right of the receptive fields. The red segments in the lines above the neural traces delineate periods of tactile stimulation; blue segments mark periods of production of a facial expression. The rasters and histograms are aligned to the start of stimulation (red dotted lines) or the start of the active facial expressions (blue dotted lines). The rasters are sorted by the duration of stimulation; for each trial the end of tactile stimulation is marked by a red dot. ***A***, A neuron with a sustained (tonic) excitatory response during stimulation of the mouth and muzzle. During bared teeth displays (fear grimaces) produced by the monkey, this neuron showed patterns of activity that resembled the external stimulation of the muzzle. ***B***, Transient, (phasic) excitatory responses to bilateral stimulation of the ears, forehead, and upper lip. This neuron also increased its firing rate during fear grimacing. ***C***, Tonic inhibitory response elicited by touching the lips and during the production of fear grimaces and in preparation to receive food or accept a nonfood item in the mouth. Other facial expressions that involved lip movements did not elicit changes in neural activity. ***D***, Tonic excitatory response to tactile stimulation anywhere on the face. This neuron did not respond during the production of facial expressions.

**Figure 2.**
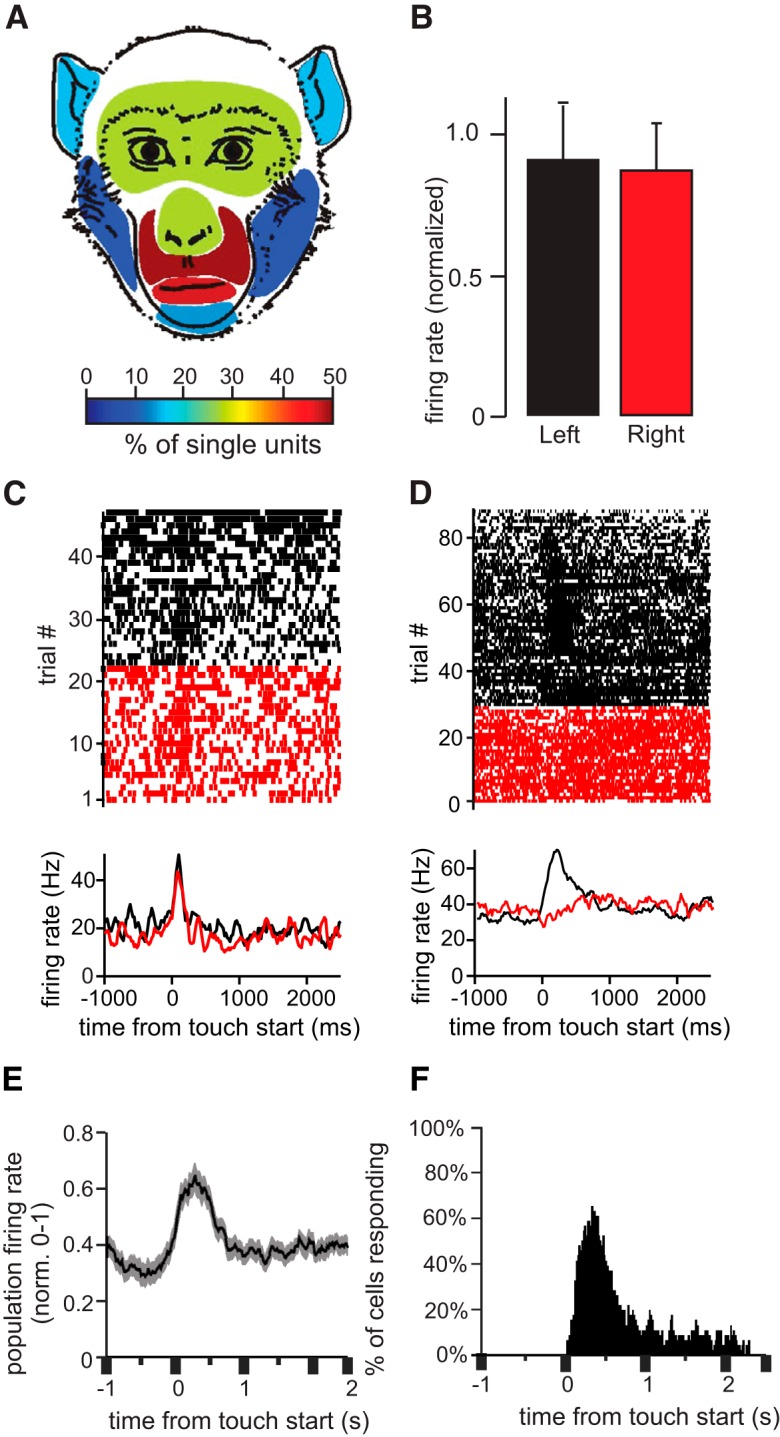
Population activity and bilateral receptive fields. ***A***, The colors in this illustration indicate the proportion of cells that responded to tactile stimulation on each facial region. Note that these values add up to more than 100% because the majority of cells responded to the touch of multiple facial regions. ***B***, Mean normalized firing rate of the population of tactile cells during stimulation of the left and right sides of the face (no significant difference, α = 0.05). ***C***, ***D***, Example neurons with bilateral (***C***) and unilateral (***D***) receptive fields. Rasters (top) and PSTHs (bottom) indicate the firing rate of the two neurons before and after tactile stimulation (zero on the *x* axis corresponds to the onset of stimulation). Trials shown in black and red correspond to stimulation of the left and right face, respectively. ***E***, Mean normalized population firing rate of all tactile cells. ***F***, Histogram showing the number of tactile cells that responded with a significant change in firing rate at each time bin following stimulation (bin size = 100 ms with 20-ms sliding window). Tactile cells modulate their firing rate throughout stimulation. The time bin at which the highest number of cells responded was 270–370 ms after stimulation onset.

Four of the 45 cells responded to tactile stimulation everywhere on the face, the remaining 41 cells responded differentially to touching different regions of the face, and 63% of the 45 neurons had composite receptive fields that included more than one spatially distinct facial feature. The receptive fields were distributed across all areas of the face, including the ears. The average receptive field size, defined as the ratio of the number of responsive face areas/number of tested face areas, was 0.477 ± 0.235 and ranged from 0.14 (1 of 7) to 1.0 (7 of 7), suggesting that the receptive fields generally were large, spanning a large proportion of the face. Figure 1 shows examples of tactile neurons and receptive fields. The receptive field of the neuron in Figure 1*A* spanned the muzzle bilaterally. The thin gray arrows inside the receptive field show a slight directional preference. Touching the muzzle elicited a sustained elevation of firing rate. In contrast, the neuron shown in Figure 1*B* showed only a transient elevation in firing rate when the muzzle, ears, or forehead was touched. This large, composite receptive field was also bilateral. The neuron shown in Figure 1*C* showed a sustained inhibition in firing rate during tactile stimulation of the lips. This neuron had one of the smallest receptive fields from the recorded population. Finally, the neuron in Figure 1*D* showed sustained elevation in firing during touch anywhere on the face. The combined receptive fields of the recorded population of 45 neurons spanned the entire face (Fig. 2*A*). Overall, the oral and perioral regions were the most strongly represented facial regions; they elicited responses in 71% of the cells (Fig. 2*A*; note that in Fig. 2, the total proportion seems to exceed 100% because as described above, almost all the recorded cells responded to touches to more than one area of the face). Although we recorded exclusively from the right amygdala, all neurons except one (Fig. 2*D*) had symmetrical, bilateral receptive fields (i.e., responded with similar changes in firing rate when the left and the right side of the face were touched; Fig. 2*B*,*C*).

### Firing rate characteristics of tactile neurons in the amygdala

Tactile neurons in the amygdala exhibited both tonic (58%) and phasic (42%) response profiles (Fig. 1*A*,*C*,*D* and 1*B*, respectively). A large portion of tactile neurons (82%) responded to stimulation with increasing firing rates (e. g., Fig. 1*A*,*B*,*D*); the rest of the recorded neurons (18%) responded with decreasing firing rates (e.g., Fig. 1*C*). The firing rate changes from baseline to maximal response ranged from 23.3 to 84.3 Hz.

### Tactile neurons respond during facial movement and the production of facial expressions

We were able to maintain stable neurophysiological recordings for seven of 45 tactile cells throughout tactile stimulation and during the production of overt facial expressions. Six of these seven neurons showed significant changes in firing rate during facial expressions (Fig. 1*A*,*B*,*C*, right side of each panel). The changes in firing rate elicited by the production of facial expressions and during touch by an external stimulus exhibited the same polarity (excitatory or inhibitory). Facial expressions were elicited by visual stimuli (such as a dangling stuffed animals with large eyes, the face of an experimenter, or the face of another monkey shown on the monitor of a handheld device in front of the monkey). Several observations support our conclusion that the firing changes of the tactile cells were due to the production of facial expressions and not to the eliciting visual stimuli. (1) The stimuli that elicited facial expressions were most effective during the first presentation and became less reliable during subsequent presentations; under these conditions the tactile neurons responded only when the monkey made a facial expression. (2) Tactile neurons responded reliably during the production of the facial expression even when the same expression was elicited by different visual stimuli. (3) The observed changes in firing rate were time-locked to the facial movements and not to the presentation of the visual stimuli.

**Figure 3.**
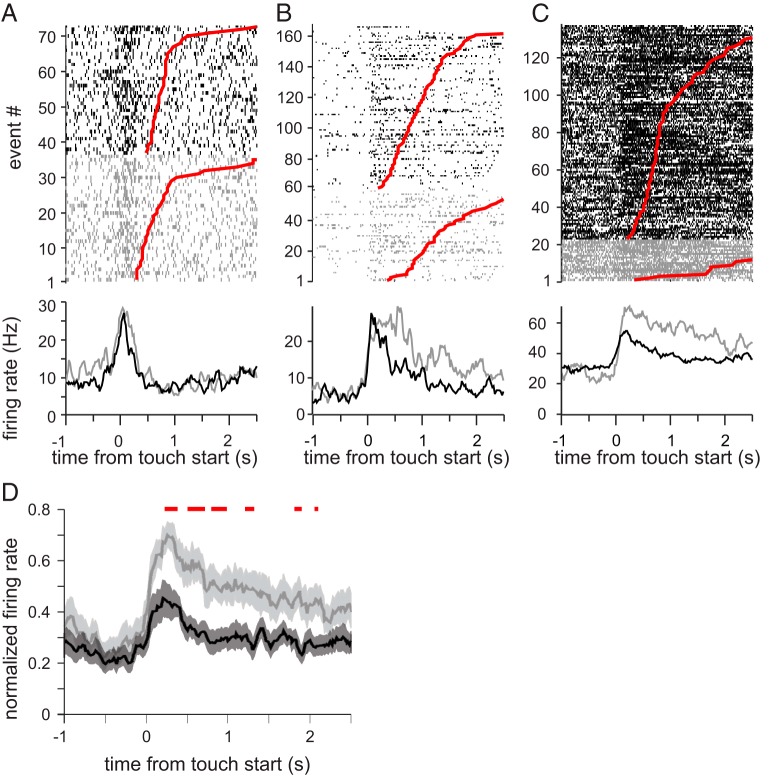
The combined effects of tactile stimulation and facial movement. The top three panels show rasters (top) and PSTHs (bottom) for the three example cells. Neural activity elicited by touch alone is shown in black, whereas neural activity elicited by touch and movement is shown in gray. On each raster, the end of the tactile stimulation is indicated by the red dot. The touch events are sorted by duration. ***A***, no difference in firing rate during touch alone and touch that elicited facial movement. ***B***, increased response duration for touches that elicited facial movement. ***C***, increased firing rate for touches that elicited facial movment. ***D***, Comparison of the normalized firing elicited in the same population of neurons by touch alone (black) and touch combined with facial movements (gray). In general, tactile stimulation combined with facial expressions led to higher firing rates. The red bar above the population histogram indicated the bins in which the firing rates are significantly different (Wilcoxon rank-sum test, α = 0.001)

Touching the face elicited facial movements on 16.1% of trials. These movements rarely amounted to full facial expressions (we have documented only nine cases of fear grimaces and threats); the rest of the time, these movements were a slight contraction of the underlying muscles. Most frequently, lip movements were elicited by the monkey attempting to orally explore the object that was touching his lips or muzzle. Some neurons had similar firing rates during touch trials and touch + movement trials (Fig. 3*A*). A subset of neurons, however, showed additional increases during touch + movement trials (Fig. 3*B*,*C*). These observations suggest that the responses of tactile neurons in the amygdala are driven by the activation of the mechanoceptors in the facial skin, and these mechanoceptors respond similarly to touch and to stretches or compressions of the skin during the contraction of underlying muscles. Furthermore, as shown in Figure 3*B*, when the tactile stimulus elicited facial movement, the duration of elevated firing rates was prolonged beyond the duration of the touch stimulus and returned to baseline only when the facial muscles relaxed. Across the population of touch-responsive cells, firing rate was doubled during touch stimuli that were accompanied by facial movement (Fig. 3*D*).

### Localization of tactile neurons in the amygdala

**Figure 4.**
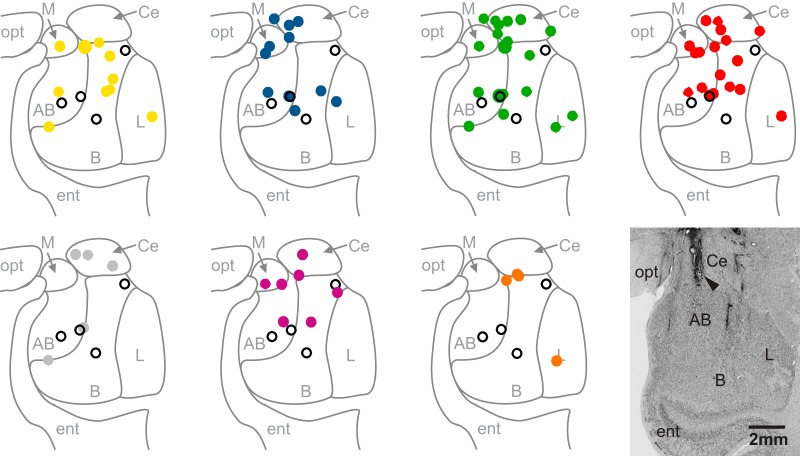
MRI-based reconstruction of recording sites. Filled circles mark the location of tactile neurons on a representative coronal section through the amygdala and are color-coded for the location of their receptive fields (yellow = nose, blue = eye region, green = muzzle, red = mouth, gray = chin, purple = ear, orange = cheek). Open circles represent neurons with large receptive fields that covered the entire face and are shown on every panel. Neurons with multiple receptive fields are shown in each panel that corresponds to their receptive fields. Histological verification of electrode location in the amygdala is from monkey Q. The arrowhead indicates an electrolytic lesion applied to the site where the neuron shown in Figure 1C was recorded. opt, optic tract; ent, entorhinal cortex; L, lateral nucleus; B, basal nucleus; AB, accessory basal nucleus; Ce, central nucleus; M, medial nucleus).

Tactile-responsive neurons were recorded from all major nuclei of the amygdala (Fig. 4). Neurons that responded to tactile stimulation of a particular facial region were found in multiple nuclei without any obvious pattern of clustering to individual nuclei.

## Discussion

We report here that a large fraction (34%) of neurons in the monkey amygdala respond to tactile stimulation of the face. Neural responses to tactile stimuli in the monkey amygdala have not been previously reported. Earlier single-unit recordings in anesthetized (Schütze et al., 1987) and awake (Knuepfer et al., 1995) cats indicated that some neurons in the amygdala were multisensory and responded to both touch and viscerosensory signals. Similarly, multisensory neurons were later found in the amygdalae of rats by Romanski et al. (1993), who demonstrated that auditory and tactile inputs converge at single neurons in the lateral nucleus of the amygdala. A role of the amygdala in touch has also been suggested by Murray and Mishkin (1983), who reported that monkeys with bilateral amygdala lesions showed deficits in tactile learning. Albeit, their impairments could not be uniquely attributed to the amygdala, as the hippocampi, entorhinal cortex, and perirhinal cortex were also damaged by the lesion. The advent of neuroimaging and a growing emphasis on touch in the framework of social neuroscience has renewed interest in the connection between the amygdala and the sense of touch. Recent neuroimaging studies have outlined many of the pathways involved in emotional touch and have shown that the amygdala is a key component of these pathways (Morrison et al., 2010; Gordon et al., 2013; McGlone et al., 2014). However, the cellular basis for this role has not yet been firmly established.

The response properties of the tactile neurons reported here resemble the properties of visually responsive neurons in the monkey amygdala. In response to both visual and tactile stimuli, neurons in the amygdala showed either phasic or tonic increases or decreases of firing rates (Mosher et al., 2010). Much like other neurons in the amygdala that evaluate the emotional significance (or value) of visual inputs (Paton et al., 2006; Morrison and Salzman, 2010; Gore et al., 2015), the tactile neurons might evaluate the inherent valence or social significance of the tactile stimuli (Gothard et al., 2007; Laine et al., 2009; Mosher et al., 2014). Future experiments specifically designed to measure neural responses to the emotional and social dimension of touch stimuli are needed to evaluate this hypothesis.

Recent neuroimaging and behavioral studies indicate that the amygdala appears to extract affective aspects of touch (e.g., McGlone and Reilly, 2010; Morrison et al., 2010; Lucas et al., 2015). Recently, touch-sensitive neurons have been reported in the monkey insula (Grandi and Gerbella, 2016). These neurons respond to sweeping stimuli that approximate how monkeys groom during social interactions. Sweeping movements of low velocity are known to activate the recently discovered tactile C fibers in the skin specialized to signal pleasurable touch (Olausson et al., 2010). The insula is a secondary somatosensory area that processes both external (environmental) and internal (visceral) signals and is highly connected to the amygdala (Aggleton et al., 1980; Friedman et al., 1986; Amaral and Insausti, 1992; Craig, 2003; Berlucchi and Aglioti, 2010; Jezzini et al., 2015; Nieuwenhuys, 2012). Projections from the insula target both the basolateral and centromedial group of amygdala nuclei. This is unusual, because cortical inputs that carry signals from the external environment typically arrive to the lateral and basal nuclei, whereas subcortical inputs from the autonomic centers in the brainstem and hypothalamus target the central nuclei (Amaral et al., 1992). By virtue of these inputs, the amygdala is well positioned to process information about both the external environment (i.e., monitoring the status of others) and the internal environment (i.e., monitoring the status of self).

We found that tactile neurons in the amygdala are activated not only when the experimenter manually stimulates the skin but also when the skin is stretched or compressed during the production of facial expressions. As the facial musculature lacks muscle spindles and tendon organs (Happak et al., 1988), the cutaneous mechanoceptors of the skin are the main source of proprioceptive feedback from the face (Cattaneo and Pavesi, 2014). Because each facial expression in the macaque is associated with a particular configuration of the facial skin (Waller et al., 2008; Parr et al., 2010), it seems possible that distinctive patterns of activity across populations of tactile neurons carry information about the facial expression of self. Such information, then, might be relayed to the amygdala to help monitor facial expression of self, as suggested by Livneh et al. (2012). It may also mediate the emotional evaluation of somatosensory inputs, similar to the well-established role of the amygdala in evaluating the emotional significance and social salience of stimuli of all sensory modalities.

## Synthesis

Reviewing Editor: Tatiana Pasternak, University of Rochester <br /> Decisions are customarily a result of the Reviewing Editor and the peer reviewers coming together and discussing their recommendations until a consensus is reached. When revisions are invited, a fact-based synthesis statement explaining their decision and outlining what is needed to prepare a revision will be listed below. The following reviewer(s) agreed to reveal their identity: Rony Paz

This paper for provides the first evidence of neurons in primate amygdala responding to the touch of faces and that the activity of these touch-responsive neurons can be modulated during facial expressions. The authors propose that these neurons may participate in extracting affective information of touch stimuli, enabling the amygdala to monitor facial expressions during social communication. Both reviewers stressed the novelty of the study and its uniqueness. However, they raised a number of issues that should be addressed before the paper can be accepted for publication in eNeuro. These are listed below.

1. Please respond to the concern about the "blindfolded" approach that prevents the visual input but does not rule out the possibility of animals producing facial expressions that are not visible to the experiments. Responses of amygdala neurons to facial touch should be distinguishable from the production of facial expressions.

2. A related point is whether the monkeys move their faces in response to touch. This could be ruled out by the analysis of the videos.

3. Please respond to the concern that responses recorded in the amygdala are to the production of facial expressions rather than to social visual stimuli. The reviewer suggests that the authors try to use temporal locking "to the expression rather than to the provocation".

4. Provide information about the selectivity of individual neurons to specific locations of stimulation. The reviewer suggests "some quantification of the receptive field", to show whether responses shown in figs 1and 2 are homogenous across all regions in the receptive field. It is also recommended that the authors include in fig 1 an example of activity in response to the stimulation outside of the receptive field.

5. The paper would also be strengthened by the inclusion in fig 2 the averaged population PSTH

6. Please clarify how the correction for multiple bins and multiple regions was done

7. In "less emphasis has been placed on auditory responses in the primate amygdala (Kuraoka and Nakamura...", add a citation of Resnik et al, Nat. Neurosci. 2016.

8. Please correct the citation of Plakke and Romanski (2016) following the suggestion of Reviewer 2.

9. As suggested by Reviewer 2, the Introduction should refer to the relevant rodent studies.

10. Please respond to the comment by Reviewer 2 on the statistical analysis described in the section "Statistical identification of receptive field' starting on line 128.

## References

[B1] Adolphs R (2010) Conceptual challenges and directions for social neuroscience. Neuron 65:752–767. 10.1016/j.neuron.2010.03.006 20346753 PMC2887730

[B2] Aggleton JP, Burton MJ, Passingham RE (1980) Cortical and subcortical afferents to the amygdala of the rhesus monkey (Macaca mulatta). Brain Res 190:347–368. 10.1016/0006-8993(80)90279-6 6768425

[B3] Amaral DG, Insausti R (1992) Retrograde transport of D-[3H]-aspartate injected into the monkey amygdaloid complex. Exp Brain Res 88:375–388. 1374347 10.1007/BF02259113

[B4] Amaral DG, Price JL (1984) Amygdalo-cortical projections in the monkey (Macaca fascicularis). J Comp Neurol 230:465–496. 10.1002/cne.902300402 6520247

[B5] Amaral, D.G., Price, J.L., Pitkanen, A., and Carmichael, T.S. (1992). Anatomical organization of the primate amygdaloid complex In The Amygdala: Neurobiological Aspects of Emotion, Memory, and Mental Dysfunction, (New York: Wiley-Liss, INC), pp. 1–66.

[B6] Berlucchi G, Aglioti SM (2010) The body in the brain revisited. Exp Brain Res 200:25–35. 10.1007/s00221-009-1970-7 19690846

[B7] Cattaneo L, Pavesi G (2014) The facial motor system. Neurosci Biobehav Rev 38:135–159. 10.1016/j.neubiorev.2013.11.002 24239732

[B8] Cho YT, Ernst M, Fudge JL (2013) Cortico-amygdala-striatal circuits are organized as hierarchical subsystems through the primate amygdala. J Neurosci Off J Soc Neurosci 33:14017–14030. 10.1523/JNEUROSCI.0170-13.2013 23986238 PMC3756751

[B9] Craig AD (2003) Interoception: the sense of the physiological condition of the body. Curr Opin Neurobiol 13:500–505. 12965300 10.1016/s0959-4388(03)00090-4

[B10] Friedman DP, Murray EA, O’Neill JB, Mishkin M (1986) Cortical connections of the somatosensory fields of the lateral sulcus of macaques: evidence for a corticolimbic pathway for touch. J Comp Neurol 252:323–347. 10.1002/cne.902520304 3793980

[B11] Gordon I, Voos AC, Bennett RH, Bolling DZ, Pelphrey KA, Kaiser MD (2013) Brain mechanisms for processing affective touch. Hum Brain Mapp 34:914–922. 10.1002/hbm.21480 22125232 PMC6869848

[B12] Gore F, Schwartz EC, Brangers BC, Aladi S, Stujenske JM, Likhtik E, Russo MJ, Gordon JA, Salzman CD, Axel R (2015) Neural representations of unconditioned stimuli in basolateral amygdala mediate innate and learned responses. Cell 162:134–145. 10.1016/j.cell.2015.06.027 26140594 PMC4526462

[B13] Gothard KM, Battaglia FP, Erickson CA, Spitler KM, Amaral DG (2007) Neural responses to facial expression and face identity in the monkey amygdala. J Neurophysiol 97:1671–1683. 10.1152/jn.00714.2006 17093126

[B14] Grandi LC, Gerbella M (2016) Single neurons in the insular cortex of a macaque monkey respond to skin brushing: preliminary data of the possible representation of pleasant touch. Front Behav Neurosci 90:doi: 10.3389/fnbeh.2016.0009 PMC4877530 27252631

[B15] Happak W, Burggasser G, Gruber H (1988) Histochemical characteristics of human mimic muscles. J Neurol Sci 83:25–35. 10.1016/0022-510X(88)90017-2 2964514

[B16] Jezzini, A., Rozzi, S., Borra, E., Gallese, V., Caruana, F., and Gerbella, M. (2015). A shared neural network for emotional expression and perception: an anatomical study in the macaque monkey. Front Behav Neurosci 9, 285–286. 26441573 10.3389/fnbeh.2015.00243 PMC4585325

[B17] Kaas JH (1993) The functional organization of somatosensory cortex in primates. Ann Anat Anat Anz Off Organ Anat Ges 175:509–518. 8297039 10.1016/s0940-9602(11)80212-8

[B18] Kennerley SW, Dahmubed AF, Lara AH, Wallis JD (2009) Neurons in the frontal lobe encode the value of multiple decision variables. J Cogn Neurosci 21:1162–1178. 10.1162/jocn.2009.21100 18752411 PMC2715848

[B19] Knuepfer MM, Eismann A, Schütze I, Stumpf H, and, Stock G (1995) Responses of single neurons in amygdala to interoceptive and exteroceptive stimuli in conscious cats. Am J Physiol 268:R666–R675. 7900909 10.1152/ajpregu.1995.268.3.R666

[B20] Kuraoka K, Nakamura K (2007) Responses of single neurons in monkey amygdala to facial and vocal emotions. J Neurophysiol 97:1379–1387. 10.1152/jn.00464.2006 17182913

[B21] Laine CM, Spitler KM, Mosher CP, Gothard KM (2009) Behavioral triggers of skin conductance responses and their neural correlates in the primate amygdala. J Neurophysiol 101:1749–1754. 10.1152/jn.91110.2008 19144740 PMC2695635

[B22] Livneh U, Resnik J, Shohat Y, Paz R (2012) Self-monitoring of social facial expressions in the primate amygdala and cingulate cortex. Proc Natl Acad Sci U S A 109:18956–18961. 10.1073/pnas.1207662109 23112157 PMC3503171

[B23] Lucas MV, Anderson LC, Bolling DZ, Pelphrey KA, Kaiser M2D (2015) Dissociating the neural correlates of experiencing and imagining affective touch. Cereb Cortex 25:2623–2630. 10.1093/cercor/bhu061 24700583 PMC4537425

[B24] McGlone F, Reilly D (2010) The cutaneous sensory system. Neurosci Biobehav Rev 34:148–159. 10.1016/j.neubiorev.2009.08.004 19712693

[B25] McGlone F, Wessberg J, Olausson H (2014) Discriminative and affective touch: sensing and feeling. Neuron 82:737–755. 10.1016/j.neuron.2014.05.001 24853935

[B26] Morrison I (2016) ALE meta-analysis reveals dissociable networks for affective and discriminative aspects of touch. Hum Brain Mapp 37:1308–1320. 10.1002/hbm.23103 26873519 PMC5066805

[B27] Morrison I, Löken LS, Olausson H (2010) The skin as a social organ. Exp Brain Res 204:305–314. 10.1007/s00221-009-2007-y 19771420

[B28] Morrison SE, Salzman CD (2010) Re-valuing the amygdala. Curr Opin Neurobiol 20:221–230. 10.1016/j.conb.2010.02.007 20299204 PMC2862774

[B29] Mosher CP, Zimmerman PE, Gothard KM (2010) Response characteristics of basolateral and centromedial neurons in the primate amygdala. J Neurosci Off J Soc Neurosci 30:16197–16207. 10.1523/JNEUROSCI.3225-10.2010 21123566 PMC3075807

[B30] Mosher CP, Zimmerman PE, Gothard KM (2014) Neurons in the monkey amygdala detect eye contact during naturalistic social interactions. Curr Biol 24:2459–2464. 10.1016/j.cub.2014.08.063 25283782 PMC4253056

[B31] Murray EA, Mishkin M (1983) Severe tactual memory deficits in monkeys after combined removal of the amygdala and hippocampus. Brain Res 270:340–344. 10.1016/0006-8993(83)90610-8 6883103

[B32] Nieuwenhuys R (2012) The insular cortex: a review. Prog Brain Res 195:123–163. 10.1016/B978-0-444-53860-4.00007-6 22230626

[B33] Olausson H, Wessberg J, Morrison I, McGlone F, Vallbo A (2010) The neurophysiology of unmyelinated tactile afferents. Neurosci Biobehav Rev 34:185–191. 10.1016/j.neubiorev.2008.09.011 18952123

[B34] Parr LA, Waller BM, Burrows AM, Gothard KM, Vick SJ (2010) Brief communication: MaqFACS: a muscle-based facial movement coding system for the rhesus macaque. Am J Phys Anthropol 143:625–630. 10.1002/ajpa.21401 20872742 PMC2988871

[B35] Paton JJ, Belova MA, Morrison SE, Salzman CD (2006) The primate amygdala represents the positive and negative value of visual stimuli during learning. Nature 439:865–870. 10.1038/nature04490 16482160 PMC2396495

[B36] Plakke B, Romanski LM (2016) Neural circuits in auditory and audiovisual memory. Brain Res 1640:278–288. 10.1016/j.brainres.2015.11.042 26656069 PMC4868791

[B37] Price JL, Amaral DG (1981) An autoradiographic study of the projections of the central nucleus of the monkey amygdala. J Neurosci Off J Soc Neurosci 1:1242–1259. 6171630 10.1523/JNEUROSCI.01-11-01242.1981 PMC6564217

[B38] Resnik J, Paz R (2015) Fear generalization in the primate amygdala. Nat Neurosci 18:188–190. 10.1038/nn.3900 25531573

[B39] Romanski LM, Clugnet MC, Bordi F, LeDoux JE (1993) Somatosensory and auditory convergence in the lateral nucleus of the amygdala. Behav Neurosci 107:444–450. 10.1037/0735-7044.107.3.444 8329134

[B40] Rudebeck PH, Mitz AR, Chacko RV, Murray EA (2013) Effects of amygdala lesions on reward-value coding in orbital and medial prefrontal cortex. Neuron 80:1519–1531. 10.1016/j.neuron.2013.09.036 24360550 PMC3872005

[B41] Rutishauser U, Tudusciuc O, Neumann D, Mamelak AN, Heller AC, Ross IB, Philpott L, Sutherling WW, Adolphs R (2011) Single-unit responses selective for whole faces in the human amygdala. Curr Biol 21:1654–1660. 10.1016/j.cub.2011.08.035 21962712 PMC4574690

[B42] Schütze I, Knuepfer MM, Eismann A, Stumpf H, and, Stock G (1987) Sensory input to single neurons in the amygdala of the cat. Exp Neurol 97:499–515. 3622705 10.1016/0014-4886(87)90109-9

[B43] Spitler KM, Gothard KM (2008) A removable silicone elastomer seal reduces granulation tissue growth and maintains the sterility of recording chambers for primate neurophysiology. J Neurosci Methods 169:23–26. 10.1016/j.jneumeth.2007.11.026 18241928 PMC2291023

[B44] Tomasi D, Volkow ND (2011) Association between functional connectivity hubs and brain networks. Cereb Cortex 21:2003–2013. 10.1093/cercor/bhq268 21282318 PMC3165965

[B45] Waller BM, Parr LA, Gothard KM, Burrows AM, Fuglevand AJ (2008) Mapping the contribution of single muscles to facial movements in the rhesus macaque. Physiol Behav 95:93–100. 10.1016/j.physbeh.2008.05.002 18582909 PMC2637410

[B46] Windels F, Yan S, Stratton PG, Sullivan R, Crane JW, Sah P (2016) Auditory tones and foot-shock recapitulate spontaneous sub-threshold activity in basolateral amygdala principal neurons and interneurons. PLoS One 11:doi: 10.1371/journal.pone.0155192 PMC4865267 27171164

